# Reactive oxygen species-mediated therapeutic response and resistance in glioblastoma

**DOI:** 10.1038/cddis.2014.566

**Published:** 2015-01-15

**Authors:** E Singer, J Judkins, N Salomonis, L Matlaf, P Soteropoulos, S McAllister, L Soroceanu

**Affiliations:** 1 California Pacific Medical Center Research Institute, San Francisco, CA, USA; 2 Cincinnati Children's Hospital Medical Center, Cincinnati, OH, USA; 3 Center for Applied Genomics, UMDNJ, Newark, NJ, USA

## Abstract

Glioblastoma (GBM) resistance to therapy is the most common cause of tumor recurrence, which is ultimately fatal in 90% of the patients 5 years after initial diagnosis. A sub-population of tumor cells with stem-like properties, glioma stem cells (GSCs), is specifically endowed to resist or adapt to the standard therapies, leading to therapeutic resistance. Several anticancer agents, collectively termed redox therapeutics, act by increasing intracellular levels of reactive oxygen species (ROS). In this study, we investigated mechanisms underlying GSC response and resistance to cannabidiol (CBD), a non-toxic, non-psychoactive cannabinoid and redox modulator. Using primary GSCs, we showed that CBD induced a robust increase in ROS, which led to the inhibition of cell survival, phosphorylated (p)-AKT, self-renewal and a significant increase in the survival of GSC-bearing mice. Inhibition of self-renewal was mediated by the activation of the p-p38 pathway and downregulation of key stem cell regulators Sox2, Id1 and p-STAT3. Following CBD treatment, a subset of GSC successfully adapted, leading to tumor regrowth. Microarray, Taqman and functional assays revealed that therapeutic resistance was mediated by enhanced expression of the antioxidant response system Xc catalytic subunit xCT (SLC7A11 (solute carrier family 7 (anionic amino-acid transporter light chain), member 11)) and ROS-dependent upregulation of mesenchymal (MES) markers with concomitant downregulation of proneural (PN) markers, also known as PN–MES transition. This ‘reprogramming' of GSCs occurred in culture and *in vivo* and was partially due to activation of the *NFE2L2* (NRF2 (nuclear factor, erythroid 2-like)) transcriptional network. Using genetic knockdown and pharmacological inhibitors of SLC7A11, we demonstrated that combining CBD treatment with the inhibition of system Xc resulted in synergistic ROS increase leading to robust antitumor effects, that is, decreased GSC survival, self-renewal, and invasion. Our investigation provides novel mechanistic insights into the antitumor activity of redox therapeutics and suggests that combinatorial approaches using small molecule modulators of ROS offer therapeutic benefits in GBM.

Glioblastoma (GBM) is the most common primary brain tumor in adults and poses significant therapeutic challenges. Recent transcriptome profiling of GBM tissues yielded molecular subclasses driven by specific genetic alterations and which correlated with patient outcome.^1, 2, 3, 4^ Among the four GBM subtypes (classical, neural, proneural (PN), and mesenchymal (MES)), MES identity is the hallmark of glioma aggressiveness and strongly associated with the poor outcome of patients.^5^ In fact, upon disease recurrence, a therapy-induced PN–MES transition (PMT) of GBM tumors has been documented in some patient samples.^5^ PMT may represent for GBM the equivalent of epithelial–MES transition associated with other aggressive cancers; however, the molecular mechanisms underlying this transition remain elusive.^6^ A subset of GBM cells with stem-like characteristics, termed glioma stem cells (GSCs), have been shown to underlie the therapeutic resistance and tumor recurrence in GBM.^6, 7^ Uncovering the mechanisms underlying the therapeutic response and resistance of GSCs is of critical importance.

Reactive oxygen species (ROS) are natural by-products of aerobic metabolism and they can promote normal cell proliferation through the activation of growth-related signaling pathways.^8^ Most anticancer drugs kill their target cells, at least in part, through the generation of elevated amounts of intracellular ROS.^9^ ROS can exert different effects according to the basal metabolic rate of the cell. The high basal metabolic rate of cancer cells makes them more susceptible to redox-directed therapeutics in comparison with non-transformed cells.^10^ Redox-directed therapeutics have been developed to act as direct inhibitors of cancer and to sensitize tumors to first-line agents; however, they are associated with significant toxicity.^9^ The discovery of non-toxic molecules that selectively upregulate ROS in malignant cells would be beneficial.

Cannabidiol (CBD) is a non-toxic and non-psychoactive cannabinoid that has been shown to have antitumor activity in multiple cancer types.^11^ Activation of CB_1_ and CB_2_ receptors has been previously shown to lead to the inhibition of tumor progression;^12^ however, CBD does not interact efficiently with CB_1_ and CB_2_ receptors, and the initial site CBD interacts with to produce antitumor activity is unknown. Our recent study demonstrated CBD-produced robust antitumor activity against a human-derived GBM in an intracranial xenograft model;^13^ however, no investigations to date have interrogated the therapeutic effects of CBD on GSCs.

One of the major systems used by both normal and cancerous cells to counteract oxidative insult is the NRF2 (also known as *NFE2L2*) transcriptionally regulated program.^9^ The role of NRF2 transcriptional regulator and SLC7A11 (solute carrier family 7 (anionic amino-acid transporter light chain), member 11) in mediating GBM response and resistance to redox-directed therapeutics has not been evaluated.

In the current study, we systematically interrogated therapeutic response to CBD using several GSC lines in culture and *in vivo*. Our results demonstrate that while CBD exhibits significant antitumor activity, a subset of GSCs adapt by activating an extended antioxidant cellular response. We show that cotargeting GSCs using ROS modulators (CBD) and inhibitors of the antioxidant response genes together is more effective than either approach alone in halting GBM growth.

## Results

### CBD inhibits GSC viability in an ROS-dependent manner

**Figure 1:**
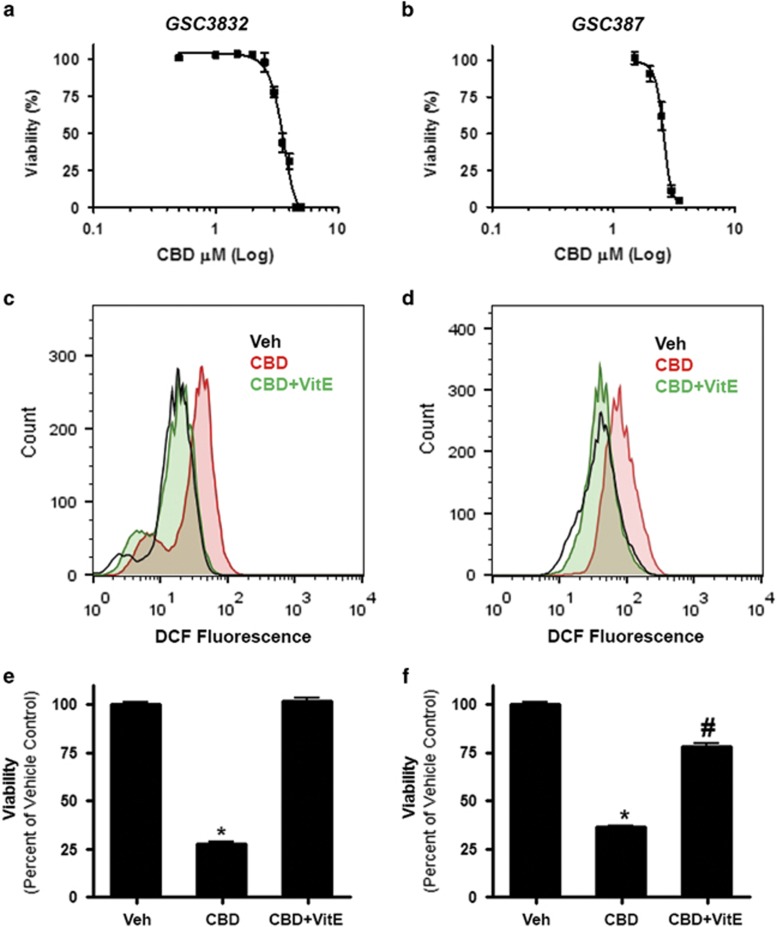
CBD inhibits GSC viability and induces apoptosis through the production of ROS. (**a** and **b**) GSC lines 3832 and 387 were treated with vehicle or CBD (*μ*M) for 2 days and cell viability was then evaluated. Viability (%) was calculated as the Dojindo reagent product absorbance in the treated cells/control cellsx100. GSCs (**c**) 3832 and (**d**) 387 were treated with vehicle or CBD (2 *μ*M) for 2 days and the production of ROS was measured using DCF and cell flow cytometry. (**e** and **f**) The contribution of ROS in CBD-dependent (2 *μ*M) reductions in cell viability was evaluated using 40 *μ*M VitE as an antagonist. Data were compared using a one-way analysis of variance (ANOVA) with a corresponding Dunnett's *post hoc* test. *^,#^Statistically significant differences from control and CBD, respectively (*P*<0.01)

Primary GSC lines 3832 and 387 were treated with a range of CBD concentrations and evaluated in viability assays. CBD inhibited the viability of 3832 and 387 GSCs with an IC_50_ (50% inhibitory concentration) value of 3.5 *μ*M (3.4–3.6) and 2.6 *μ*M (2.5–2.7), respectively (Figures 1a and b). In both GSC cultures, CBD increased the production of ROS (Figures 1c and d), which was reversed in the presence of vitamin E (VitE) (or *α*-tocopherol). In agreement with these data, the effects of CBD on cell viability were reversed in the presence of the ROS scavenger (Figures 1e and f).

### CBD treatment improves survival of mice bearing intracranial GSC xenografts

**Figure 2:**
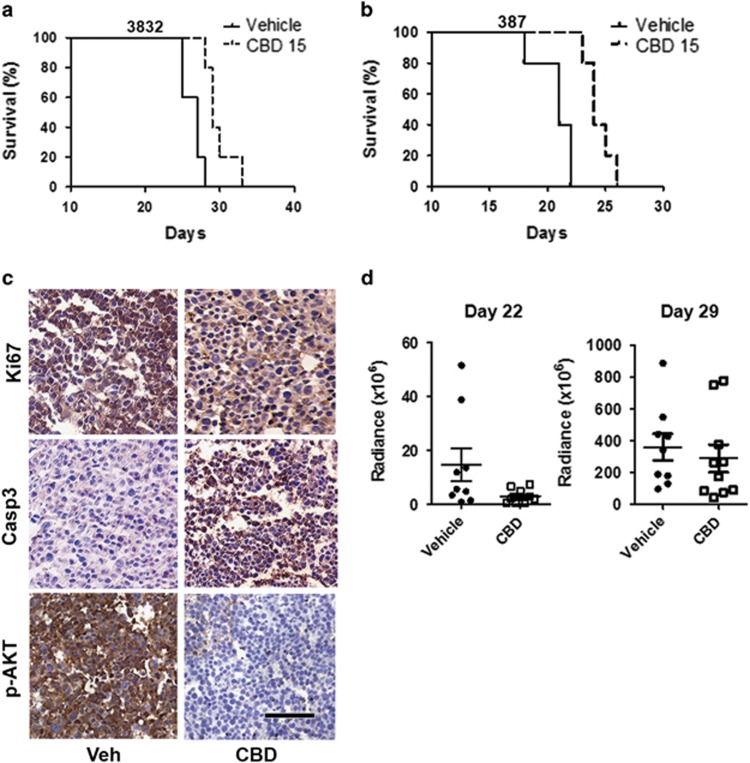
CBD prolongs survival mice bearing intracranial tumors derived from GSCs. Tumors were generated in female athymic nu/nu mice by the intracranial injection of 5 × 10^3^ cells (**a**) 3832 or (**b**) 387 luciferase-labeled cells in 4 *μ*l of media. Treatments were started at day 9 following BLI confirmation of tumor presence (using IVIS Lumina) and randomization of mice. Starting on day 9, 15 mg/kg of CBD was administered intraperitoneally 5 days a week until completion of the experiment. Animal health was monitored daily and when mice showed signs of disease progression, they were humanely killed in accordance with institutional IACUC guidelines. Survival data was evaluated using Kaplan–Meier curves and a long-rank Mantel–Cox test. (**c**) Representative tumor sections represent inhibition of Ki67 (upper panel) staining, upregulation of cleaved caspase-3 (middle panel) and inhibition of p-AKT (lower panel) induced by CBD. Tumors were harvested 25 days after tumor induction. Bar=200 *μ*m. (**d**) BLI measurements demonstrate initial response to CBD (reduction of tumor size at day 22), followed by tumor resistance to treatment 1 week later (day 29)

We next performed efficacy studies *in vivo*, using two models of intracranial GBM xenografts, established by a low number (5 × 10^3^) of GSC lines 3832 and 387. As demonstrated in Figure 2, this low number of cells can seed highly aggressive tumors. Tumor progression is rapid and the median survival in 3832 and 387 cells was 27 and 21 days, respectively. CBD treatment significantly prolonged survival in tumor bearing mice (Figures 2a and b). Immunohistochemical (IHC) analyses of GBM xenograft tissues demonstrated that CBD treatment inhibited p-AKT, Ki67 and stimulated the activation of caspase-3 in GBM *in vivo* (Figure 2c). Control antibody and hematoxylin and eosin staining are shown in Supplementary Figure 2. Using bioluminescence measurements, we monitored tumor growth and response to CBD therapy in real time. Our data demonstrate that following initial inhibition of tumor growth by CBD (day 22), intracranial GBM tumors appear to resume a more rapid growth rate in spite of continuous CBD administration (Figure 2d). These data suggest that *in vivo*, a sub-population of CBD-treated GSCs adapted during therapy and became resistant. We next set out to investigate mechanisms underlying both response and resistance to CBD treatment in tumors derived from GSCs.

### CBD inhibits GSC self-renewal in an ROS-dependent manner

**Figure 3:**
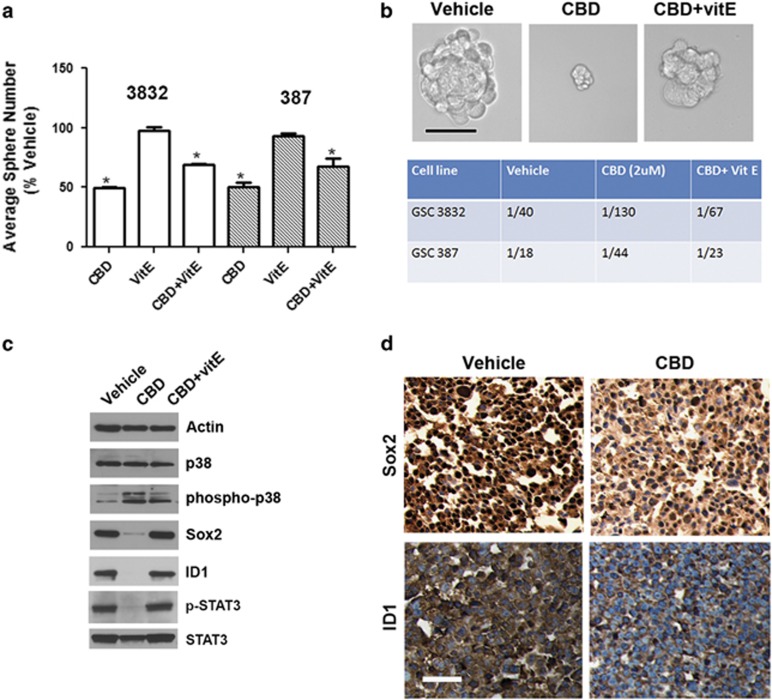
CBD inhibits GSC self-renewal. (**a**) GSC lines 3832 and 387 were subjected to sphere formation assays and sphere numbers were recorded 10 days later. *^, #^*P*<0.01 for 2 *μ*M CBD and 2 *μ*M CBD+40 *μ*M VitE, respectively, compared with vehicle. The experiment was repeated two times. (**b**) Sphere frequency was measured using a limited dilution assay in both GSC lines treated with CBD±VitE. Upper panel shows representative photomicrographs in the indicated conditions. Lower panel table shows median stem cell frequency calculated using the ELDA software. (**c**) Western blot analyses in 3832 cells treated with 2 *μ*M CBD or CBD+VitE for 48 h, using the indicated antibodies. Activation of the p-p38 pathway concomitantly with the downregulation of the self-renewal master regulators, p-STAT3, Sox2 and Id1, are shown. (**d**) IHC analyses of xenograft glioma tissues from mice treated with vehicle or CBD (22 days). Staining with Sox2 and ID1 antibodies are shown. Bar= 100 *μ*m

We previously showed that CBD inhibits Id1 expression in primary-derived GBM lines and that Id1 genetic knockdown inhibits self-renewal and Sox2 expression in primary GBM cells.^14^ In this study, we directly investigated the effects of CBD on self-renewal and stemness of GSC. Using sphere formation assays (10, 100 and 1000 cells per well), we determined that CBD inhibits GSC self-renewal in both 3832 and 387 lines and this effect was partially reversed by cotreatment with the antioxidant VitE (40 *μ*M) (Figures 3a and b). Using the limited dilution tumorsphere formation assay in conjunction with extreme limiting dilution analysis (ELDA) software analysis package (available from Walter+Eliza Hall Institute, Parkville, VIC, Australia),^15^ we determined that the cancer stem cell frequency was significantly inhibited by CBD in both GSC lines (Figure 3b, lower panel). Mechanistically, we found that CBD inhibits the expression of Sox2, Id1, p-STAT3 and upregulates phosphorylated (p)-p38 MAPK, all of which have been connected to inhibition of self-renewal and stemness in GBM.^16^ Importantly, these effects were also dependent on ROS production, as demonstrated by partial or complete reversal induced by cotreatment with VitE (Figure 3c). IHC analyses of GBM xenograft tissues demonstrated that CBD treatment inhibited both Id1 and Sox2 expression *in vivo* (Figure 3d).

### CBD treatment upregulates antioxidant response genes and induces a shift to an MES molecular phenotype

**Figure 4:**
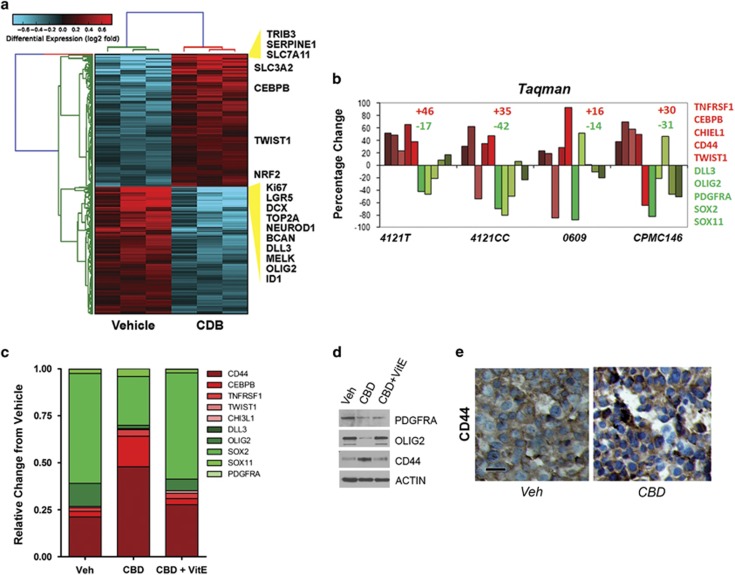
CBD treatment induces ROS-dependent PMT in GBM. (**a**) CBD treatment results in large-scale differential gene expression. Gene expression as log 2 fold changes, normalized between vehicle- and CBD- (2 *μ*M) treated groups for each cell line, are shown for 2107 protein coding and 243 noncoding RNAs (fold >1.25 and false discovery rates (FDR)-adjusted *t*-test, *P*<0.1). Predicted crucial regulatory factors are indicated on the right of the graph. Red depicts upregulation, while blue indicates downregulation. (**b**) Taqman validation of several PN (indicated in green) and MES (in red) markers in three additional GSC lines and an acutely dissociated patient tissue (CPMC146) confirm CBD-induced PMT. (**c**) Taqman analysis of PN and MES markers in 3832 cells treated with CBD or CBD+VitE. Results were normalized to Rab14 expression levels. (**d**) The 3832 GSCs treated with CBD±VitE were subjected to western blot analysis using the indicated antibodies to detect PN (Olig2) and MES (CD44) markers. (**e**) IHC analysis of GBM xenograft tissue sections from mice treated with vehicle or CBD (intraperitoneally 15 mg/kg, 5 days per week). Tumor tissue was harvested at 29 days after tumor implantation. Bar=50 *μ*m

To investigate the mechanism underlying the resistant GBM phenotype suggested by partial therapeutic response (Figures 2c and d), we profiled RNA extracted from three GSC lines treated with vehicle and CBD on Affymetrix Gene St 1 DNA Arrays (Affymetrix, Inc., Santa Clara, CA, USA) (Figure 4a). Raw data is available at http://www.ncbi.nlm.nih.gov/geo/query/acc.cgi?acc=GSE57978.

Using standard analyses of microarray in conjunction with Altanalyze software (developed by Dr. Nathan Salomonis; http://www.genmapp.org/AltAnalyze/), we determined that significantly altered transcripts belong to several functional classes. Specifically, we measured a downregulation of stemness markers (MELK, OLIG2), PN markers (DLL3, PDGFRA) and proliferation markers (Ki67, Top2A) (Figure 4a). Concomitantly, we measured significant upregulation of several antioxidant response gene products (SLC7A11, NRF2) as well as MES GBM markers (CD44, TNSFR10, CEBPB; Figure 4a). Taqman validation for a subset of 12 genes was performed using additional GSC lines, as well as acutely dissociated patient GBM cells treated with CBD. Taqman analysis confirmed the molecular signature shift induced by CBD (Figure 4b). Western blot analysis further corroborated these findings in both 3832 and 387 GSC lines (Supplementary Figure 1). Taken together, these data suggest that the partial therapeutic efficacy of CBD against GBM is due to a subset of tumor cells upregulating antioxidant response genes and undergoing an adaptive reprogramming toward a resistant, MES phenotype. PMT has been previously documented to occur in GBM following radiation^5^or antiangiogenic therapy.^17^ Given the significant upregulation of the antioxidant response gene products, we investigated whether the phenotypic shift toward a resistant, MES molecular profile was ROS-dependent. Cotreatment with ROS scavenger VitE partially reverted the CBD-induced MES shift measured in 3832 GSCs both at the transcript and protein levels (Figures 4c and d). In agreement with these data, upregulation of the MES marker CD44 was measured specifically in GBM xenografts from CBD-treated mice, *in vivo* (Figure 4e).

### CBD-induced antioxidant response is mediated by NRF2 activation

**Figure 5:**
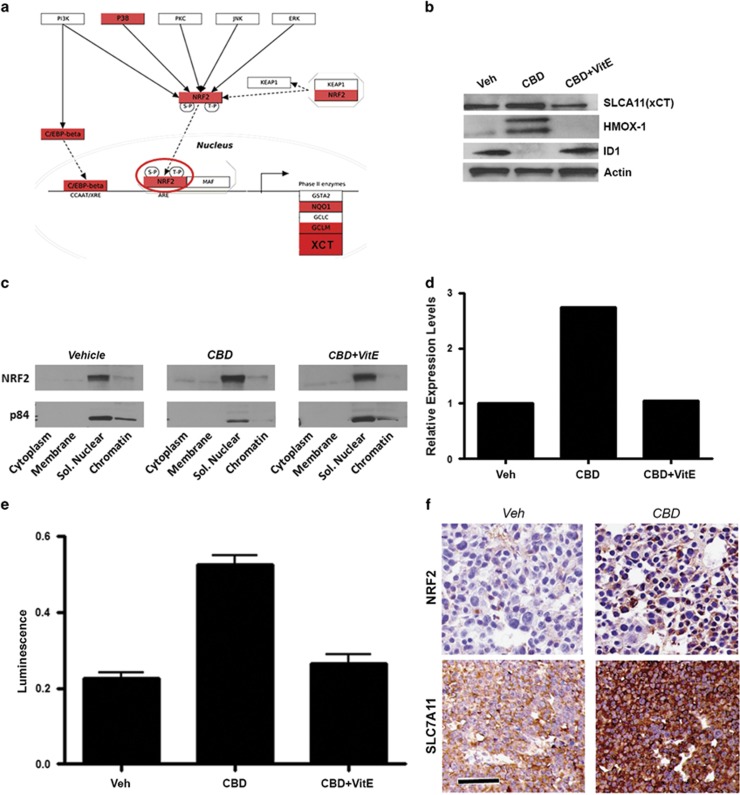
CBD induces nuclear translocation and activation of NRF2 and its downstream targets. (**a**) Visualization of CBD-associated gene expression changes along the NRF2 transcriptional network. Molecules shown in red were significantly upregulated by CBD (>2 × ). (**b**) Western blot analysis confirms ROS-dependent upregulation of NRF2 target proteins HMOX-1 and xCT, following 48 h treatment with CBD±40 *μ*M VitE. (**c**) Subcellular fractionation using GSC 3832 cell line treated with CBD±VitE was used to generate protein lysates for western blot with the indicated antibodies. (**d**) Quantification of NRF2 levels using densitometry shows that relative to p84 (control), nuclear levels of NRF2 are enhanced by CBD treatment. (**e**) GSCs were transfected using the Qiagen Cignal Antioxidant Luciferase Reporter Kit (Qiagen Inc, Valencia, CA, USA; no. CCS-5020L) and Lipofectamine 2000 (Life Technologies) in OptiMEM media and 1% NEAA. At 24 h after transfection, the cells were treated with vehicle, 2.0 *μ*M CBD, 2.0 *μ*M CBD+40 *μ*M VitE or dl-sulforapahane (40 *μ*M) the manufacturer's recommended positive control for 48 h. NRF2 activity was measured using the Promega Dual-GLO Luciferase Assay system (Promega Corporation, Sunnyvale, CA, USA). First, NRF2-dependent luminescence was measured and then constitutively expressed luminescence was measured to normalize for cell number. The plotted luminescence units are a ratio of the two readings. Data were compared using a one-way analysis of variance (ANOVA) with a corresponding Dunnett's *post hoc* test. *^,#^Statistically significant differences from control and CBD at *P*<0.05, respectively. (**f**) IHC detection of NRF2 and SLC7A11 (xCT) in GBM xenograft tissues from mice treated with vehicle or CBD for 22 days. Bar= 200 *μ*m

Analysis of transcription factor activation, predicted from the analysis of microarray data using Altanalyze software indicated significant activation of the *NFE2L2* (NRF2) transcriptional network (Figure 5a). To confirm the activation of NRF2 in another GSC line, we used 3832 cells treated with CBD or CBD+VitE. Western blot analysis 48 h following treatment demonstrates that the expression levels of the NRF2 targets SLC7A11 (xCT) and HMOX-1 were upregulated and these effects were reversed by VitE (Figure 5b). Next, we sought to investigate the molecular mechanism underlying NRF2-mediated activation of xCT and the overall antioxidant response program induced by CBD. In resting conditions, NRF2 is held inactive in the cytoplasm by KEAP1, being translocated to the nucleus following oxidative injury or stress.^18^ We used western blot analyses of subcellular fractions from CBD-treated GSC 3832, and determined that upon CBD treatment, the nuclear fraction of NRF2 was increased, an effect reversed by VitE (Figures 5c and d). Promoter activation assays confirmed that CBD induced NRF2 activation in a concentration-dependent manner and this effect was reversed by VitE (Figure 5e). Taken together, these data indicate that CBD induces NRF2 activation, which in turns induces antioxidant response genes. Importantly, IHC analyses of xenograft tumor tissues from glioma bearing mice demonstrated *in vivo* upregulation of NRF2 and xCT in CBD treated as compared with vehicle-treated tumors (Figure 5e), suggesting that this may be a key pathway underlying resistance to the CBD-based redox therapeutic.

### Genetic targeting of SLC7A11 inhibits GSC self-renewal and cooperates with CBD to inhibit cell survival

Given the robust upregulation of SLC7A11, we hypothesized that targeting its expression or function would revert some of the adaptive antioxidant response and thus render a more efficient therapeutic response in GSCs. We next used a pool of small interfering RNA (siRNA) sequences to target SLC7A11 in GSC 387, and measured the effects on cell viability and reduced glutathione (GSH) levels. We found that SLC7A11 knockdown inhibited GSH levels and these effects were reverted by pretreatment with the *N*-acetyl cysteine antioxidant (Supplementary Figures 3a and b). SLC7A11 knockdown cells showed enhanced sensitivity to CBD as compared with control siRNA-treated GSCs (Supplementary Figure 3c). To stably inhibit the expression levels of SLC7A11, we used a lentivirus-mediated knockdown, using two distinct short hairpin RNA (shRNA) sequences. Protein knockdown was significant (Supplementary Figure 3d) and the xCT-knockdown GSCs were impaired in their ability to form tumorspheres (Supplementary Figures 3e and f). Attempts to expand xCT-knockdown cells for *in vivo* studies were not successful. As previously reported for cell lines,^19^ pharmacological inhibition of xCT inhibited reduced GSH levels in GSC 3832 (Supplementary Figure 4).

### Pharmacological targeting of xCT/system Xc inhibits GSC survival, in an additive manner with CBD

Analysis of multiple PN and MES GBM samples demonstrated MES gliomas consistently expressed higher levels of xCT (Supplementary Figure 5a). These results suggest that the upregulation of xCT occurs in more aggressive GBM tumors. We next wished to pharmacologically inhibit xCT using sulfasalazine (SAS), which has been previously shown to inhibit its function.^19^ SAS inhibited tumorsphere formation in GSCs by ~40% (Supplementary Figure 5b). Concentration–response curves showed that SAS inhibited GSC 3832 and 387 cell viability with IC_50_ values of 564 *μ*M (546–582) and 548 *μ*M (522–575), respectively, demonstrating limited potency of the drug in culture (Supplementary Figures 5c and d). At these concentrations, SAS had limited solubility. We next measured the effect of combining CBD and SAS to target GSCs. Even under suboptimal conditions (due to SAS precipitation at high concentration), the combination of SAS enhanced the activity of CBD in an additive manner (Supplementary Figures 5c and e). Western blot analysis of SAS-treated GSC cultures demonstrated inhibition of proliferation markers (TOP2A) and upregulation of xCT (Supplementary Figure 5f). Administration of SAS *in vivo* was ineffective at targeting intracranial tumors derived from GSC 3832 cells because of its limited solubility and lack of potency. As shown in Supplementary Figure 5h, the levels of SLC7A11 were not upregulated in intracranial tumors derived from GSC 3832 cells during treatment with SAS, unlike the cultured GSCs, which have direct access to SAS. Given the limitations of administering SAS at effective concentrations *in vivo* to target GSCs, we turned our attention to a recently discovered novel class of system Xc inhibitors, Erastin (ERA), and its analog, piperazine erastine (PE). Both drugs showed efficacy in inhibiting system Xc in several cancer cell lines and induced cell death via an iron-dependent mechanism, which is named as ferroptosis, in culture and in a subcutaneous xenograft cancer model.^20, 21, 22^ Although neither drug is able to cross the blood–brain barrier, their improved potency for targeting xCT made them important tools for further evaluating combination therapy in culture with our cannabinoid-based redox therapeutic, CBD.

### Novel system Xc inhibitors ERA and PE inhibit GSC viability in a dose-dependent manner and act synergistically with CBD to inhibit GSC viability

**Figure 6:**
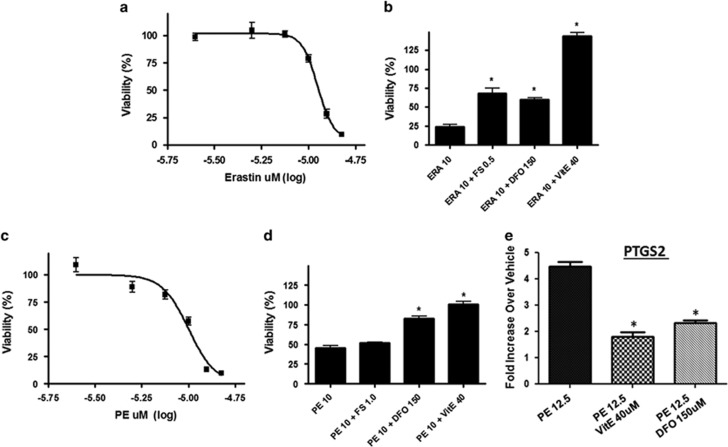
ERA and PE inhibit GSC viability in an iron-dependent manner. (**a** and **c**) GSCs 387 were treated with *μ*M concentrations of ERA or PE for 5 days and the percentage of cell viability was calculated as the Dojindo reagent product absorbance in the treated cells/control cells × 100. (**b**) GSC 387 were treated with indicated *μ*M concentrations of ERA±DFO, Fer or VitE, as indicated. (**d**) GSC 387 was treated with shown *μ*M concentrations of PE±DFO, Fer or VitE. (**e**) Taqman determination of *PTGS2* levels after GSC 387 treatment with PE±VitE and DFO. *P<0.02 ANOVA

ERA and PE inhibited GSC 387 viability with an IC_50_ value of 11.1 *μ*M (10.5–11.8) and 10 *μ*M (9.5–10.5), respectively (Figures 6a and c). In 387 cells, the inhibitory effects of ERA and PE were partially reverted by the iron chelators deferoxamine mesylate (DFO) and ferrostatin (Fer) (Figures 6b and d), demonstrating that these small molecules exert their antitumor effect along the same pathways as described for other cancers.^20, 21^ PE upregulated *PTGS2* (prostaglandin-endoperoxidase synthase 2) levels in GSC and this effect was reversible by VitE and DFO (Figure 6e). PTGS2 is a pharmacodynamic marker of system Xc inhibition.^20, 21, 22^

**Figure 7:**
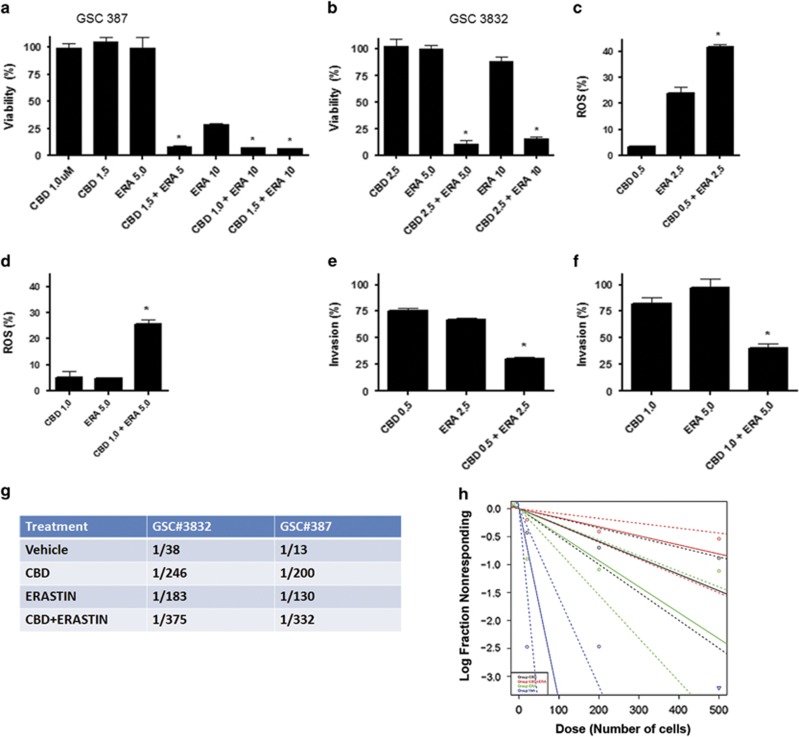
CBD and ERA synergistically inhibit GSC viability, invasion and self-renewal. (**a** and **b**) GSC lines 387 and 3832 were treated with CBD, ERA or CBD+ERA (*μ*M). Viability (%) was calculated as the Dojindo reagent product absorbance in the treated cells/control cells × 100. These data were used to calculate a CI value (0.53 and 0.49 for GSC 387, and 0.89 and 0.85 for GSC 3832) for the two combined concentrations in each group, where a CI value of <1, 1 and >1 indicates synergistic, additive and antagonistic effects, respectively (see also Supplementary Table 1). (**c** and **d**) ROS measurements show the combination of CBD+ERA (*μ*M) enhanced ROS production in the 3832 and 387 GSC lines. ROS was measured using 2′,7′dichloro-dihydrofluorescein and cell flow cytometry. The % increase in ROS was calculated as the FL2 emission shift in treated cells/vehicle cells × 100. (**e** and **f**) Invasion assays were performed using GSC 387, GSC 3832 in the presence of vehicle, CBD, ERA and combination of CBD+ERA (*μ*M). Invasion (%) was calculated as the number of cells invading in the treated cells/control cells × 100. Data were compared using a one-way analysis of variance (ANOVA) with a corresponding Dunnett's *post hoc* test. *Statistically significant differences from control at *P*<0.05. (**g** and **h**) Limited dilution assays were performed using 3832 and 387 GSCs cultured in three decreasing cell densities. Inhibitors were added at the time of initial culturing (CBD=1.5 *μ*M, ERA=5 *μ*M). The number of wells with spheres was recorded 10 days following initial culturing and data were analyzed using ELDA software. The plot represents analysis for 387 cells; the chart includes estimated (median) stem cell frequency for both GSC lines in the presence of CBD, ERA and CBD+ERA (*μ*M) as indicated. Overall test for stem cell frequency between any of the groups shown in (**g** and **h**) had a *P*-value of 2.2e−5

Importantly, CBD in combination with ERA led to a synergistic increase in the inhibition of GSC viability (Figures 7a and b). The effect on viability with the combination of CBD+ERA correlated with an enhanced production of ROS (Figures 7c and d). Furthermore, we performed two functional assays, which measure pathognomonic features of glioma, that is, invasion and self-renewal. Matrigel invasion assays demonstrate that CBD and ERA synergistically inhibited tumor cell invasion, as shown in Figures 7e and f. Self-renewal was assessed using a limited dilution assay, which demonstrated that treatment with CBD+ERA significantly downregulated GBM stem cell frequency in two GSC cell lines (Figures 7g and h). The combination treatment was more efficient than either drug used alone (Figures 7g and h). We have also tested the combination of PE and CBD on GSC 387 viability and our results demonstrate that the two drugs act synergistically to inhibit tumor cell viability (Supplementary Figure 6). Supplementary Table 1 summarizes combination index (CI) values for CBD and various system Xc inhibitors in inhibiting GSC survival, where a CI value of <1, 1 and >1 indicates synergistic, additive and antagonistic effects, respectively.

## Discussion

To study the response of GSC to a redox therapeutic that stimulates ROS, we used the non-toxic, non-psychoactive cannabinoid, CBD. CBD has been previously shown to inhibit GBM progression in subcutaneous and intracranial models representing bulk tumor mass.^14, 23, 24^ CBD has also been shown to inhibit the progression of other cancers *in vivo*.^25, 26, 27, 28^ The most unifying mode of action in culture for these effects has been the selective production of ROS in tumor cells as opposed to non-transformed cells.^11^ However, whether the ability of CBD to inhibit cancer aggressive through the production of ROS in culture is linked to the antitumor activity of CBD *in vivo* has not been established.

In this investigation, CBD-dependent inhibition of GSC viability in culture was mediated by an increase in the production of ROS, which could be reverted by the antioxidant VitE.

CBD-induced therapeutic benefit can be attributed, in part, to the inhibition of GSC self-renewal and stemness as shown by functional assays and biomarker analyses in xenograft tissues. Our results confirm the recent report that p-p38 activation underlies ROS-mediated reprogramming of GSCs^29^ and furthermore show for the first time that stem cell key regulators such as Id1, Sox2 and p-STAT3 are inhibited by CBD in an ROS-dependent manner. These findings are relevant in the context of using CBD as adjuvant therapy for GBM, to complement the standard of care (e.g., Temodar, radiation), which is not efficacious against GSCs.^30^

CBD was able to inhibit GBM progression *in vivo* and prolonged survival. IHC analysis of tumor tissue demonstrated CBD-dependent inhibition of cancer cell proliferation and induction of apoptosis, as assessed by decreased Ki67 and increased cleaved caspase-3 staining, respectively. Overall, however, the inhibitory effects on GSC progression *in vivo* were not as robust as when using standard glioma lines such as U87^23^ and U251^13^ cells. This provided us with an excellent opportunity to understand GSC adaptive changes to a redox therapeutic.

Using DNA microarrays, we interrogated changes occurring in GSCs following CBD treatment. Data analysis demonstrated that CBD significantly inhibited the expression of stem cell regulators and GBM PN markers, with a concomitant upregulation of antioxidant response gene products and MES markers. Using western blot assays, we discovered that induction of the antioxidant response and the MES phenotype were both reversible by antioxidant pretreatment, suggesting a dependence on ROS. More specifically, we measured the activation of a critical antioxidant response element (ARE) activator, NRF2. Upon sensing of increase ROS levels, NRF2 is released from its complex with KEAP1, and it translocates to the nucleus, where it binds ARE found in the promoters of ROS-detoxifying enzymes, including NQO1, GST, HMOX-1 and xCT.^18, 31^ Importantly, augmented expression of both NRF2 and xCT was observed *in vivo* in CBD-treated tumor tissue, as compared with vehicle. This demonstrates for the first time that CBD targeting of GSC results in the activation of pathways responsible for counteracting oxidative stress *in vivo*.

Cysteine metabolism, regulated by xCT, is a critical determinant of GBM growth and invasion. Interestingly, recent studies have identified xCT as a key regulator of cancer cell metabolic reprogramming and therapeutic target in gastric cancer and in triple-negative breast carcinomas.^32, 33^ In this study, we explored the utility of small-molecule inhibitors of xCT as antiglioma agents. SAS, previously used in other published reports,^19^ exhibited limited antitumor activity as monotherapy, and acted in an additive manner with CBD. Because of its low potency and limited solubility, combination experiments in xenograft models could not be performed. We next tested a new generation of system Xc inhibitors, ERA and PE. ERA has been identified through a screen for small molecules efficacious against Ras-driven cancers and modulates intracellular levels of GSH and ROS.^34^ This is the first study to report the activity of ERA against primary GSCs. In other cancers, cell killing by ERA occurs as a novel iron-dependent, cellular death mechanism, which is named as ferroptosis.^21^ Our studies using specific iron chelators demonstrate that in GBM cells, ERA works akin to inhibition of other cancers. As ERA has low solubility in organic solvents, PE has been recently synthesized and characterized as an alternative to ERA for targeting tumor cells.^21^ We showed that PE is efficacious against GBM cells and this effect can be partially reverted by iron chelators. Importantly, both ERA and PE act synergistically with CBD to inhibit viability of GSC. However, unlike the case of flank tumors, PE does not cross the blood–brain barrier and as such was not suitable for *in vivo* testing in our orthotopic GBM model. Novel PE derivatives with better blood barrier penetrance and alternate delivery approaches are currently under investigation.

**Figure 8:**
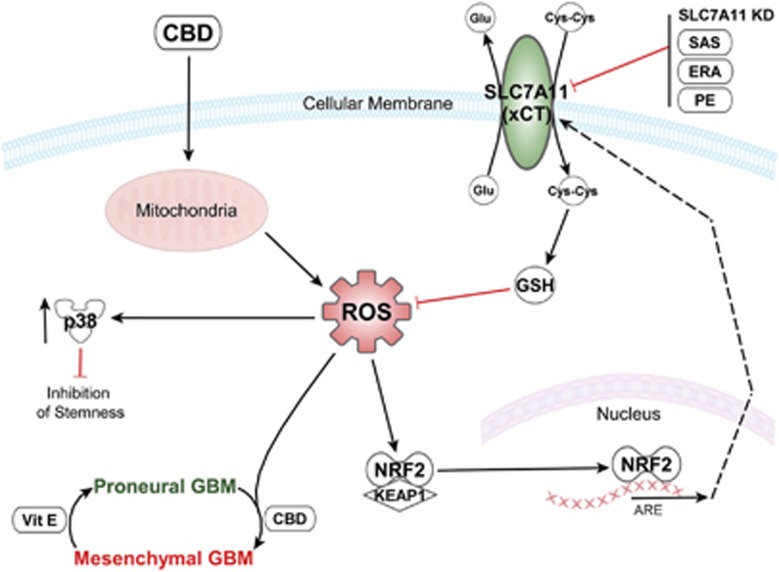
Diagram summarizes adaptive signaling pathways activated by CBD via ROS upregulation (PMT and antioxidant response, both reversible) in GSCs. Cotargeting using CBD and system Xc (xCT) inhibitors synergistically inhibit GSC viability. The diagram was produced using the Ingenuity Pathway Analysis software

Our data presented herein demonstrates for the first time that administration of CBD can inhibit intracranial growth of primary GSC-derived tumors *in vivo* and this effect is mediated by an increase in ROS levels. Our results support the notion that CB-based therapeutics in combination with other non-toxic small-molecule inhibitors of antioxidant response genes can synergistically inhibit GBM progression and should be considered for the development of novel therapeutics. The diagram in Figure 8 summarizes adaptive signaling pathways modulated by CBD via ROS up-regulation in glioma stem cells.

## Materials and Methods

### Cell lines and reagents

The U251 cell line was obtained from ATCC (Manassas, VA, USA). GSC lines 387 and 3832 were generously provided by Dr Jeremy Rich, while all other GSC lines used were generated in-house from tissue samples obtained during surgical resection of patients diagnosed with GBM, using an IRB-approved protocol. Tumors were then subjected to enzymatic digest, mechanically dissociated and cultured as neurospheres as previously described by our group. Tumor lines were maintained as subcutaneous flank xenografts in athymic Nu/Nu mice and processed as stated above. All GSC lines were cultured in growth media made up of Neurobasal Media (Lifetech, Chicago, IL, USA) supplemented with N2 (Lifetech), GlutMAX (Lifetech), basic fibroblast growth factor and epidermal growth factor, both at 25 *μ*g/ml (Promega Corp., Madison, WI, USA), unless stated otherwise. CBD was obtained from the National Institutes of Health through the National Institute of Drug Abuse. Ethanol served as the vehicle control in all culture studies. Tocopherol (TOC; Sigma-Aldrich, St. Louis, MO, USA), SAS (Sigma-Aldrich), Fer (Sigma-Aldrich), DFO (Abcam Ltd, Boston, MA, USA) and ERA (Selleck Chemicals, Houston, TX, USA) were all obtained commercially from indicated vendors. PE was generously donated by Dr. Brent Stockwell (Columbia University, New York, NY, USA).

### Cellular ROS detection using flow cytometry

ROS measurements were made by plating GBM cells at 0.175 × 10^6^ cells per well in 6-well plates precoated with GelTrex Reduced Growth Factor Basement Membrane Matrix (Life Technologies, Grand Island, NY, USA) for 3 h at 0.04–0.06 mg/ml (dependent on the manufacturer's stated concentration range). Cells were allowed to recover overnight. At 24 h, media were aspirated and replaced with fresh growth media lacking GlutMAX (Lifetech) and supplemented with indicated drug concentrations and the addition of 2′-7′ dichlorofluorescein diacetate (DCF; Sigma-Aldrich) at 10 *μ*M. Cultures were incubated with treatment media for 12 h, harvested with TrypLE Express (Lifetech), resuspended in 300 *μ*l PBS and ROS was assessed by cell flow cytometry. Representative histogram plots were made using FlowJo software (Tree Star Inc., Ashland, OR, USA).

### Expression profiling using Human Gene 1.0 ST Affymetrix Arrays

Affymetrix Human Gene 1.0 ST arrays were processed according to the Affymetrix Expression Analysis Whole Transcript (WT) Sense Target Labeling Protocol (Affymetrix, Inc.). Briefly, total RNA (300 ng) was converted to double-strand cDNA. cRNA was obtained by an *in vitro* transcription reaction and used as the template for generating a new first-strand cDNA. The cDNA was fragmented, end-labeled with biotin and hybridized to the array for 16 h at 45 °C using the GeneChip Hybridization Oven 640 (Affymetrix, Inc.). Washing and staining with streptavidin–phycoerythrin was performed using the GeneChip Fluidics Station 450 and the images acquired using the Affymetrix Scanner 3000 7G Plus (Affymetrix, Inc.). The data were normalized using quantile normalization with the RMA algorithm for gene-level intensities and the ratio determined for each gene using Partek Genomics Suite (Partek Inc., St. Louis, MO, USA). Total RNA was processed at the microarray facility from the center for applied genomics at Public Health Research Institute in New Jersey, using the Affymetrix Gene 1.0 ST Array.

### Human affymetrix data analysis

Mean values of selected human and HCMV transcripts in the IE1KD *versus* Control are displayed using the R program heatmap.2 from the package ‘gplots'. The package is available from the R repository CRAN, and is maintained by Gregory R. Warnes. Raw data is available at http://www.ncbi.nlm.nih.gov/geo/query/acc.cgi?acc=GSE57978.

Additional analyses were performed using AltAnalyze software developed by Dr Nathan Salomonis and Ingenuity analysis software (Ingenuity IPA, Redwood City, CA, USA).

### Limited dilution assays and sphere formation

CD133+ cells from primary GSC lines maintained on the flank of nude mice were plated in 96-well plates at 10, 100 and 1000 cells per well (12 wells/condition) in complete growth media. Cells were retrieved after sorting using MACS directly in Neurobasal media+growth factors and inhibitors were added directly to cells at the time of initial culturing. Tumorspheres were evaluated 10 days following initial culturing, using an inverted microscope fitted with a camera. Wells were scored positive when at least one sphere was present. The estimated stem cell frequency was estimated using the ELDA. For percent sphere formation, 100 GSCs were plated per well of a 24 well plate and sphere formation (at 7 days) was calculated using the following formula: percent sphere formation (%)=number of spheres/number of cells plated × 100. Each condition was run in quadruplicate and the experiment was repeated two times.

### Orthotopic mouse model of GBM

Tumors were generated in female athymic nu/nu mice by the intracranial injection of luciferase-labeled GSC 3832 or 387 in 4 *μ*l of RPMI. Treatments were started at day 9 (arrow) following bioluminescence imaging (BLI) confirmation of tumor presence (using IVIS Lumina instrument, Perkin Elmer, Santa Clara, CA, USA) and randomization of mice. Starting on day 9, CBD was administered intraperitoneally 5 days a week until completion of the experiment. Animal health was monitored daily, and when mice showed signs of disease progression, they were humanely killed in accordance with institutional IACUC guidelines.

### Data analysis and statistical procedures

All data shown represents two independent experiments with ≥3 replicates. The IC_50_ values with corresponding 95% confidence limits were compared by the analysis of logged data (GraphPad Prism; Graphpad Software Inc., La Jolla, CA, USA). Significant differences were also determined using a one-way ANOVA or the unpaired Student's *t*-test, where suitable. Survival data was evaluated using Kaplan–Meir curves and a log-rank Mantel–Cox test. *P*-values <0.05 defined statistical significance. Statistical significance was assessed using GraphPad Prism (Graphpad Software Inc.).

## Abbreviations

ARE: antioxidant response element
BLI: biolouminescence imaging
CBD: cannabidiol
CI: combination index
DCF: 2′,7′ dichlorofluorescein diacetate
ELDA: extreme limited dilution assay
ERA: erastin
Fer: ferrostatin
GBM: glioblastoma
GSC: glioma stem cell
GSH: glutathione (reduced)
IC_50_: 50% inhibitory concentration
IHC: immunohistochemistry
MES: mesenchymal
NRF2: nuclear factor, erythroid 2-like
PMT: proneural–mesenchymal transition
PN: proneural
PTGS2: prostaglandin-endoperoxidase synthase 2
ROS: reactive oxygen species
SAS: sulfasalazine
SLC7A11: solute carrier family 7 (anionic amino-acid transporter light chain), member 11
siRNA: small interfering RNA
shRNA: short hairpin RNA
VitE: vitamin E
