# Supplementary material for: Reactive oxygen species-mediated therapeutic response and resistance in glioblastoma
Source: Cell Death Dis. 2015 Jan 15;6(1):e1601–. doi: 10.1038/cddis.2014.566 (PMC4669764; doi:10.1038/cddis.2014.566)
Supplement: Supplementary Information [file cddis2014566x1.pdf]

Reactive Oxygen Species-mediated Therapeutic Response and Resistance in Glioblastoma

Eric Singer, Jonathon Judkins, Nathan Salomonis, Lisa Matlaf, Pat Soteropoulos, Sean McAllister* and Liliana Soroceanu*

Supplementary Information

This file includes:

Supplementary Methods

Supplementary Figures 1-6 and legends

Supplementary Table 1 and legend

Supplementary Methods

Cell viability and Apoptosis Assays

Primary GBM viability assays for dose response and cytotoxicity rescue with Tocopherol were performed by seeding cells at 10,000 cells/well in triplicates into 96-well plates containing the appropriate growth media. Cells were allowed to recover o/n, and then growth media was supplemented with the indicated drugs to the desired final concentrations. Viability was assessed at 5 days using Dojindo's Cell Counting Kit-8 (Dojindo Molecular Technologies, Inc., Rockville, MD, USA). Due to colorimetric interference of Dojoindo's CCK-8 by SAS, viability assays involving SAS were assessed at 5 days using CellTiter-GLO Luminescent Cell Viability Assay (Promega). Data shown is representative of an $n \geq 6$ for all data points, and all data analysis was performed using GraphPad Prism.

NRF2 Promoter Activity Assay

U251 cells were plated at 6,000 cells/well in a 96-well plate in RPMI 1640 (Life Technologies #11875-119) with 10% FBS and allowed to recover for 24 hours. The cells were then transfected using the Qiagen Cignal Antioxidant Luciferase Reporter Kit (Qiagen #CCS-5020L) and Lipofectamine 2000 (Life Technologies #11668-019) at a 1:200 diliution in OptiMEM media with 5% FBS and 1% NEAA. 24 hours post-transfection, the cells were treated in triplicate with EtOH, CBD 2.0 µM, CBD 2.0 µM + Tocopherol 40 µM, or DL-sulforapahane, the manufacturer's recommended positive control. After another 18 hours, NRF2 activity was measured using the Promega Dual-GLO Luciferase Assay system (Promega #E2920). First, NRF2-dependent luminescence was measured and then constitutively expressed luminescence was measured to normalize for cell number. The plotted luminescence units are a ratio of the two

readings. Data was compiled from two independent experiments (n=6) and statistical analysis was performed using a one-way ANOVA test with Tukey's Multiple Comparison post-test. There is significant difference between the Veh and CBD treatment groups ($p<.001$) as well as between the CBD and CBD + Toc groups ($p<.001$). No significant difference is present between the Veh and CBD + Toc groups ($p>.05$).

Subcellular Fractionation Western

3832 GSC were plated in six-well plates at 300,000 cells/well in neurosphere media as described above. After 24 hours, they were treated with EtOH, CBD, or CBD + Tocopherol and collected 48 hours later using the Pierce Subcellular Protein Fractionation Kit for Cultured Cells (#78840). For the Western blot, the antibodies used were Biocheck α-ID1 (#195-14), Sigma-Aldrich α-actin (A2066), Abcam α-NRF2 (#ab62352), and Genetex α-p84 (#GTX70220).

TaqMan

RNA was harvested using the Qiagen RNeasy kit and cDNA was prepared using the Biorad iScript cDNA Synthesis Kit (#170-8891) with 1ug of RNA. Taqman Fast Universal PCR Master mix (Life Technologies #4352042) was used with various Taqman Gene Expression Assays (Life Technologies). We used the Life Technologies Viia 7 Real Time PCR Machine and ran triplicates of each gene. RAB-14 (Prod # Hs00249440_m1) was used as a reference gene. Relative Quantification (ddct) method was used to perform comparative analysis of gene expression in vehicle versus treated cells.

Western Blotting

Cells were collected using RIPA buffer (Pierce #89901) with protease and phosphatase inhibitors (Pierce). Samples were quantified using the BCA Protein Assay (Pierce #23228) and BSA standards (Pierce #23210). Samples were prepared with BioRad XT Sample buffer (#161-0791) and XT Reducing agent (#161-0792). They were run on a BioRad XT 4-12% Bis-Tris gel (#345-0123) with Kaleidoscope Precision Protein Standards (BioRad #161-0375). Protein was transferred to PVDF membrane (BioRad #162-0177) and blocked in 5% skim milk or 5% BSA (Thermo #37520) for phospho-proteins. The primary antibodies used were Biocheck α-ID1 (#195-14), Sigma-Aldrich α-actin (A2066), Abcam α-SLC7A11 (ab37185) Abcam α-SLC7A5 (ab85226), Cell Signaling α-CD44 (#5604), Cell Signaling α-PDGFRα (#3174), Cell Signaling α-phospho p38 (#9211), Cell Signaling α-p38 (#9212), Millipore α-Olig2 (#Ab9610), and Epitomics α-Sox2 (#2683-1). The secondary antibodies used were Thermo anti-rabbit HRP (#32460) or Thermo anti-mouse HRP (#32430). Bands were visualized with Termo Super Signal West Femto (#34094).

Proliferation and Invasion assays

MTT assays (measuring cell proliferation/viability) and Boyden chamber invasion assays were performed as previously described [1].

Immunohistochemical analyses were conducted as previously detailed by our group (Ref. 14, main text). Primary antibodies were the same as listed for western blot analyses. Secondary reagents were all from Biogenex. Photomicrographs were acquired using a Nikon microscope fitted with a camera; images were processed using Adobe Photoshop.

RNA Interference

We used the approach previously described by our group (Ref 14) to transfect primary GSC cells with “validated” predesigned siRNA targeting SLC7A11 (and control non-targeting oligonucleotides) from Ambion (Catalog # AM16708). Functional assays were run 72-120 hours following protein knockdown.

SLC7A11 target specific shRNA expression lentiviruses were custom made by Gene Target Inc and used the following sequences:

sh Negative Control: sense: GTCTCCACGCGCAGTACATTT antisense: AAATGTACTGCGCGTGGAGAC

SLC7A11 shRNA 1; sense: CTGGGTGGAACTCCTCATAAT antisense: ATTATGAGGAGTTCCACCCAC

SLC7A11 shRNA2; sense: ACTCCTCTACCAGCTGTTATT antisense: AATAACAGCTGGTAGAGGAGT

Glutathione Measurements

Glutathione Assay kit was purchased from Cayman (Catalog # 703002) and used according to the manufacturer’s instructions using GSC cultured in 96 wells. GSH measurements were performed 48 and 72h following protein knockdown or treatment with inhibitors.

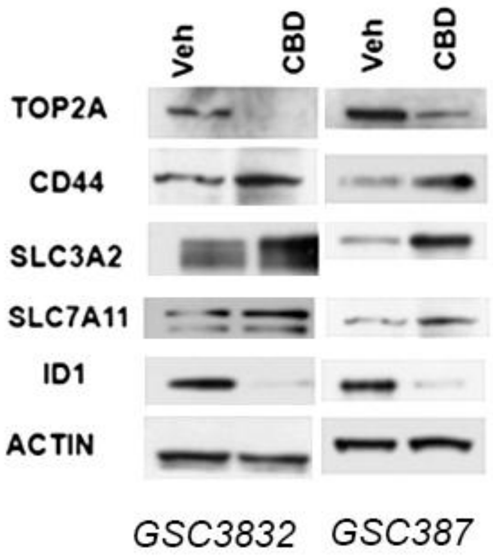

Supplementary Figure 1. **CBD treatment modulates proliferation (TOP2A), stemness (ID1), mesenchymal marker (CD44) and xCT levels in primary GBM lines.** 3832 and 387 cells treated with vehicle or CBD (2uM, 48h) were used to generate cell lysates which were subjected to western blot analysis with the indicated antibodies.

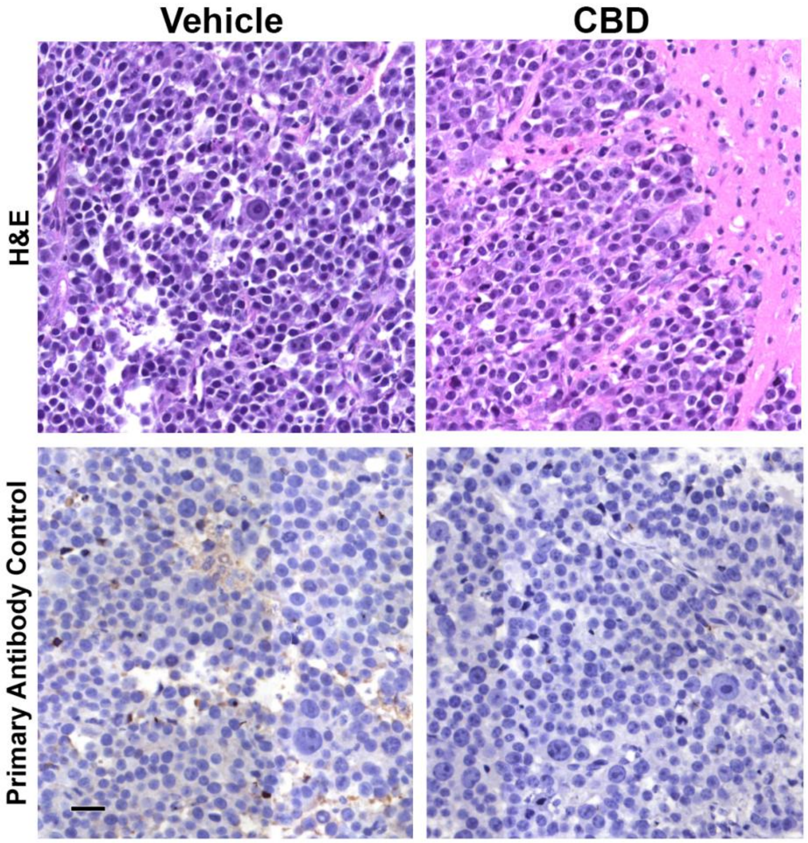

Supplementary Figure 2. **H& E and control antibody staining of GSC-derived xenograft tissues**. Serial sections from paraffin embedded GBM xenograft tissues were processed for immunohistochemical analysis for various markers. Upper panels show representative H&E staining of tumor sections from vehicle and CBD-treated mice (tissues were harvested 29 days post tumor implantation). Lower panels were stained with a rabbit IgG as a control for IHC analyses shown in Figures 2-5. Bar= 50um.

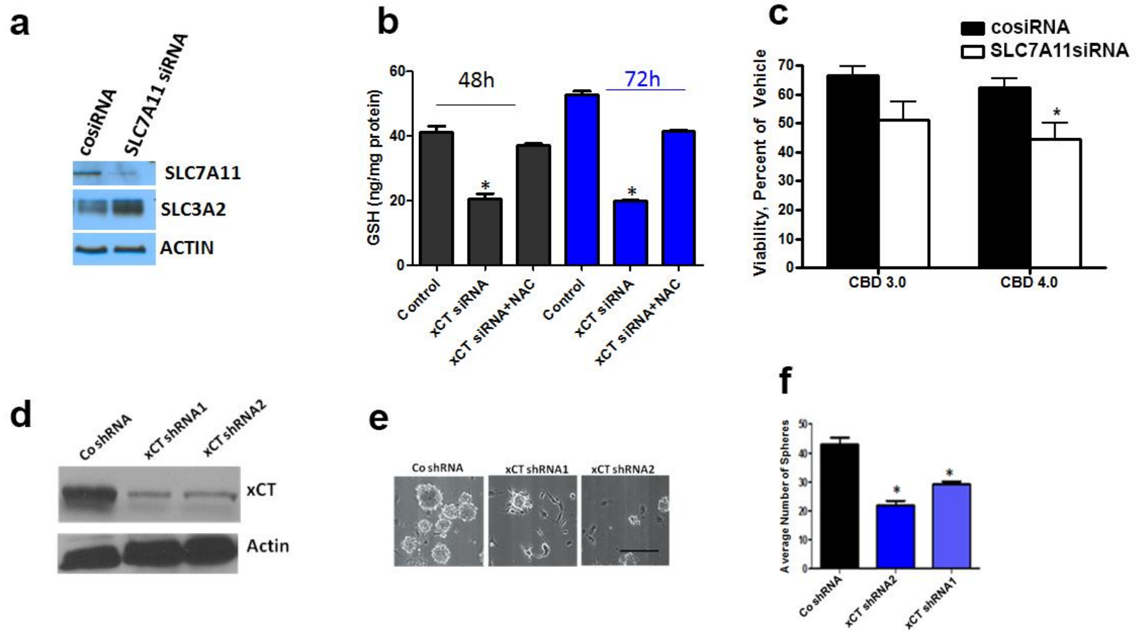

Supplementary Figure 3. **SLC7A11 (xCT) knockdown inhibits GSC viability and self-renewal**. **a**. Control siRNA and SLC7A11 (xCT) siRNA treated 3832 cells were processed for western blot analysis 72h following transfection, using the indicated antibodies. **b**. Reduced glutathione levels were measured using a kit from Cayman. siRNA treated cultures at 48h and 72h. Each condition was run in triplicate. Results show a representative experiment (repeated twice). **c**. Five day viability assays were carried out in control siRNA and SLC7A11siRNA treated 3832 cells in the presence or absence of CBD at two concentrations. Cell counts were processed as explained for Figure 7. *p=0.002, student t-Test. **d**. Two distinct short hairpain RNA oligos targeting SLC7A11 (xCT) and control were used to transfect 387 cells. Expression levels of xCT were measured five days following transfection using western blot analysis. **e**. The modified xCT knockdown GSC 387 cells were used in tumorsphere assays. **f**. Quantification of tumorsphere assays. Seven days following initial culturing. Each condition was run in quadruplicate and the experiment was repeated twice. *p<0.01, student t-Test.

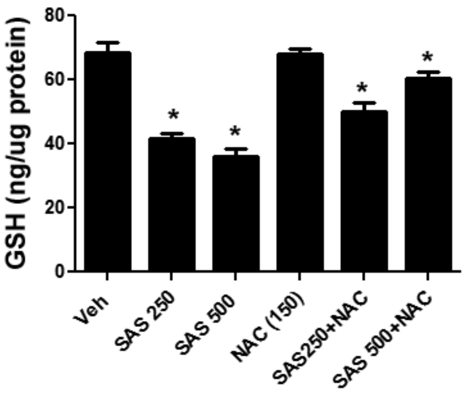

Supplementary Figure 4 . **SAS modulates GSH levels in GSC**. Glutathione levels were measured in 3832 cells, using a kit from Cayman, according to manufecturer's instructions. SAS and NAC (uM indicated) were added to samples at the time of culturing. Readout was performed 72h later. Each condition was run in triplicate. Results are from one representative experiment, out of two repeats. * $p < 0.05$ ANOVA.

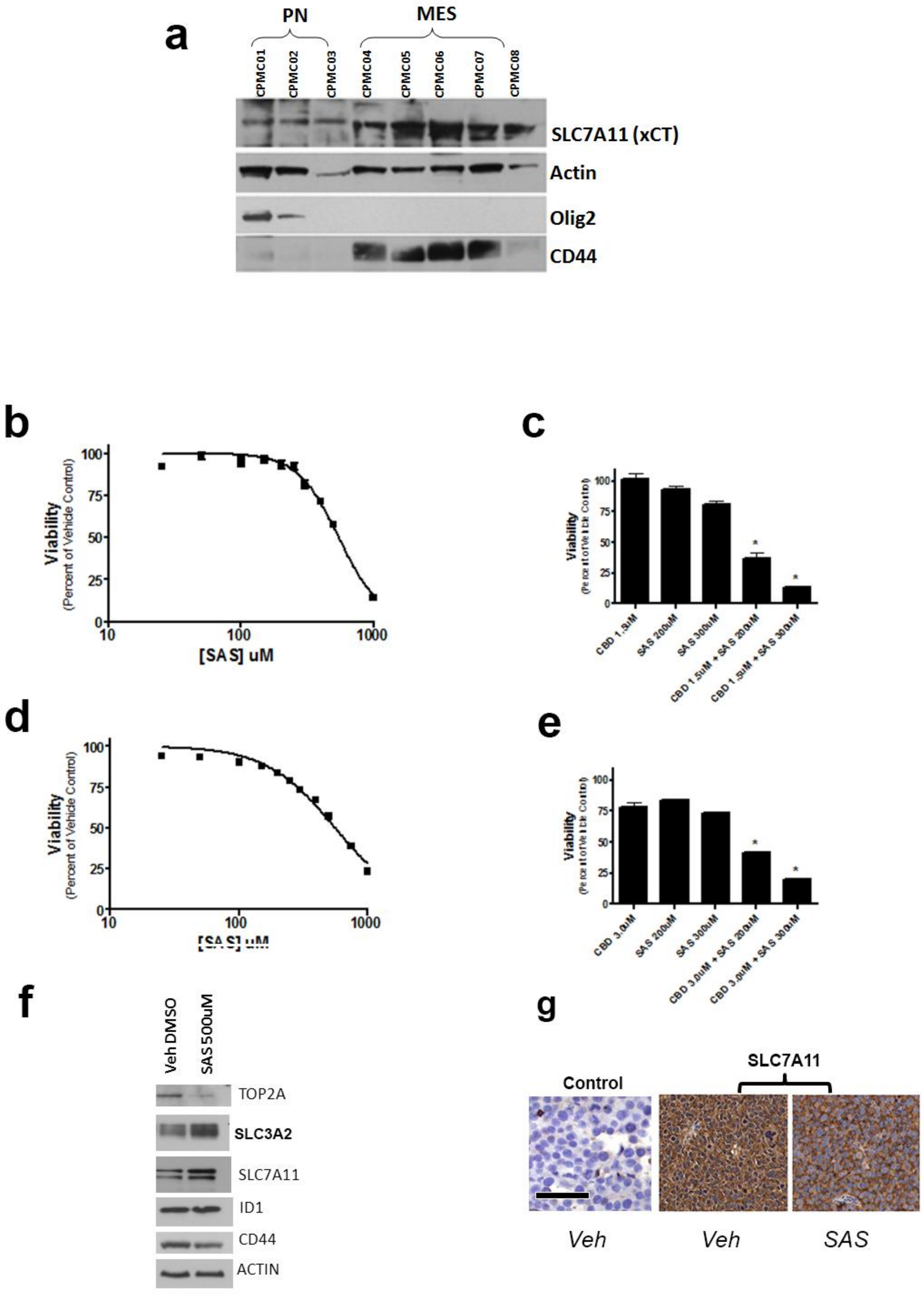
a
PN
MES
CPMC01
CPMC02
CPMC03
CPMC04
CPMC05
CPMC06
CPMC07
CPMC08
SLC7A11 (xCT)
Actin
Olig2
CD44
b
Viability
(Percent of Vehicle Control)
[SAS] uM
c
CBD 1.5uM
SAS 200uM
SAS 300uM
CBD 1.5uM + SAS 200uM
CBD 1.5uM + SAS 300uM
d
Viability
(Percent of Vehicle Control)
e
CBD 3.0uM
SAS 200uM
SAS 300uM
CBD 3.0uM + SAS 200uM
CBD 3.0uM + SAS 300uM
f
Veh DMSO
SAS 500uM
TOP2A
SLC3A2
SLC7A11
ID1
CD44
ACTIN
g
Control
SLC7A11
Veh
Veh
SAS

Supplementary Figure 5. xCT inhibition using Sulfasalazine (SAS) cooperates with CBD in inhibiting GSC viability. a. A set of proneural and mesenchymal primary GBM samples were used to measure SLC7A11 levels using western blot analysis. Levels of proneural (Olig2) and mesenchymal (CD44) markers were also measured. SLC7A11 expression is enriched in mesenchymal GBMs. **b**. Dose response curve, measuring effects of SAS on viability of GCS 3832. GSC 3832 was plated out at 10,000 cells per well in 96-well plates and allowed to recover for 24 hr. Media was then supplemented with drug or DMSO to reach the indicated final working concentrations of SAS. Viability was assessed at day five using Promega's CellTiter-GLO Luminescent Cell Viability Assay. Data represents two independent experiments with an n=6. Data analysis and non-linear regression was performed using Graphpad Prism. **c**. Viability assay data for GCS 3832 treated with SAS and CBD. Data represents two independent experiments with an n=6. Bars represent S.E.; *P<0.001. **d**. Dose response curve for SAS treatment in GCS 387. Drug treatment and data collection and analysis were performed as explained for GSC3832. **e**. Viability assay using GSC 387 treated with CBD and SAS, as explained above. Data represents two independent experiments with an n=6. Bars represent S.E.; *P<0.001. **f**. Vehicle or SAS (400uM, 48h) treated 387 cells were harvested in cell lysis buffer and subjected to western blot analysis with the indicated antibodies. **g**. Tumor bearing nude mice were treated i.p. with 80 mg/kg SAS, twice daily, 5 days/week, for 21 days following tumor confirmation by imaging (day 9). Xenograft tumor tissues from vehicle or SAS treated mice were processed for SLC7A11 immunohistochemistry. Bar= 200um.

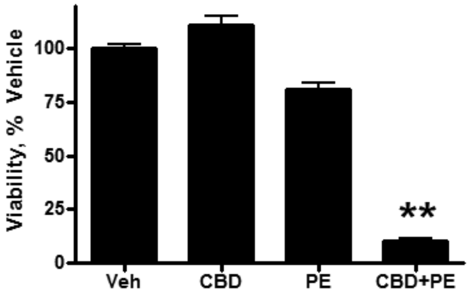

Supplementary Figure 6. **CBD and PE synergistically inhibit GSC viability.** 387 GSC were plated in 96-well plates at 10,000 cells/well in Neurobasal media supplemented with glutamine, penicillin/streptomycin, and N-2. The cells were allowed to recover for 24 hours at 37°C and then treated with piperazine erastin (PE), cannabidiol (CBD), or a combination of the two. After 120 hours (5 days), the number of viable cells was evaluated with the Dojindo Cell Counting Kit 8. Data was generated from two independent experiments. Statistical analysis was performed in GraphPad Prism using a one-way ANOVA test and Tukey's Multiple Comparison post-test. There are significant differences between CBD and the combination ($p<.001$) and PE and the combination ($p<.001$).

Supplementary Table 1. Combination Index For CBD and system Xc inhibitors

Primary line	Drug Combination	CI value	
387	CBD (1.5μM) + SAS (200μM)	0.98	387
CBD (1 μM) + SAS (300μM)	0.64		
387	CBD (1.5μM) + Erastin (5μM)	0.53	387
CBD (1 μM) + Erastin (10μM)	0.50		
3832	CBD (2.5μM) + Erastin (5μM)	0.52	3832
CBD (2.5μM) + Erastin (10μM)	0.75		

Treatments combining CBD with SAS or Erastin produce additive to synergistic inhibition of primary GBM cell viability. 387 and 3832 cells were treated for two days with vehicle (control), CBD, SAS or Erastin **(Figure 6, supplementary Fig 5)**. Specific dose ratios of CBD + SAS and CBD + Erastin where then combined in 387 and 3832 cells. Cell viability (%) was calculated as the MTT product absorbance in the treated cells/control cells x 100. These data were used to calculate CI values as previously described by our group [2]. A CI value of <1, 1, and >1 indicates synergism, additivity, and antagonism, respectively [3]. Data are the mean of at least 3 independent experiments; bars, ± SEM.

1. McAllister SD, Christian RT, Horowitz MP, Garcia A, Desprez PY. Cannabidiol as a novel inhibitor of Id-1 gene expression in aggressive breast cancer cells. *Mol Cancer Ther* 2007, **6**(11)**:** 2921-2927.

2. Marcu JP, Christian RT, Lau D, Zielinski AJ, Horowitz MP, Lee J*, et al.* Cannabidiol enhances the inhibitory effects of delta9-tetrahydrocannabinol on human glioblastoma cell proliferation and survival. *Mol Cancer Ther* 2010, **9**(1)**:** 180-189.

3. Chou TC, Tan QH, Sirotnak FM. Quantitation of the synergistic interaction of edatrexate and cisplatin in vitro. *Cancer Chemother Pharmacol* 1993, **31**(4)**:** 259-264.
